# SMART SKY EYE System for Preliminary Structural Safety Assessment of Buildings Using Unmanned Aerial Vehicles

**DOI:** 10.3390/s22072762

**Published:** 2022-04-03

**Authors:** Jaehoon Bae, Jonghoon Lee, Arum Jang, Young K. Ju, Min Jae Park

**Affiliations:** 1 Department of Architectural Design, College of Engineering Science, Chonnam National University, Gwangju 59626, Korea; skycity-bjh@jnu.ac.kr; 2 Joong Ang ENR Co., Ltd., Seoul 05645, Korea; realjh312@gmail.com; 3 School of Civil, Environmental and Architectural Engineering, Korea University, Seoul 02841, Korea; rum97@korea.ac.kr

**Keywords:** SMART SKY EYE, unmanned aerial vehicle, safety inspection, thermography, structural safety assessment

## Abstract

The development of unmanned aerial vehicles (UAVs) is expected to become one of the most commercialized research areas in the world over the next decade. Globally, unmanned aircraft have been increasingly used for safety surveillance in the construction industry and civil engineering fields. This paper presents an aerial image-based approach using UAVs to inspect cracks and deformations in buildings. A state-of-the-art safety evaluation method termed SMART SKY EYE (Smart building safety assessment system using UAV) is introduced; this system utilizes an unmanned airplane equipped with a thermal camera and programmed with various surveying efficiency improvement methods, such as thermography, machine-learning algorithms, and 3D point cloud modeling. Using this method, crack maps, crack depths, and the deformations of structures can be obtained. Error rates are compared between the proposed and conventional methods.

## 1. Introduction

In recent years, there has been a growing interest in emerging information technologies, such as unmanned aerial vehicles (UAVs), artificial intelligence (AI), and big data, and these technologies are applied in various existing industries to increase productivity. According to the Teal group [1], the civil unmanned aerial systems field will undergo dynamic growth and commercialization in the next generation. In addition, the construction industry is expected to become highly invigorated with the help of advanced technologies, which have a high potential in construction and facility maintenance. Several studies have investigated the potential applications of UAVs on construction sites [2,3,4]. 

Siebert and Teizer [2] used UAVs to measure the conditions of the infrastructures. For utilizing the three-dimensional (3D) mapping data obtained through UAV, a photogrammetry program and a program for generating a point cloud are described. In addition, a performance model for estimating positional errors was tested in the actual construction site. Goessens et al. [3] investigated UAVs for robotizing construction sites. Laboratory tests with a large UAV showed the feasibility of constructing future real scale structures capable of transporting building elements and assembling them. Similarly, Hallermann and Morgenthal [4] performed aerial photography of chimneys over 200 m in height and the UAV detected concrete cracks. They discussed the application of UAVs with high-definition photographs and video cameras, which confirmed the possibility of structural safety testing. Khaloo and Lattanzi [5] used 3D modeling aerial photographs to find defects in structures.

The Pohang earthquake, which struck the northern region of South Korea in 2017, with a magnitude of 5.4, raised awareness about the importance of rapid safety inspections of buildings in affected areas. Conventional damage assessment of buildings has been dependent on human resources and specialized equipment, with exterior surveys of buildings being conducted through visual inspection, which involves costs and time restrictions on checking the diverse range of damages and obtaining objective visualized data. Additionally, as buildings become taller, this traditional building inspection method requires additional time and expenses to ensure inspector safety [6]. 

**Figure 1:**
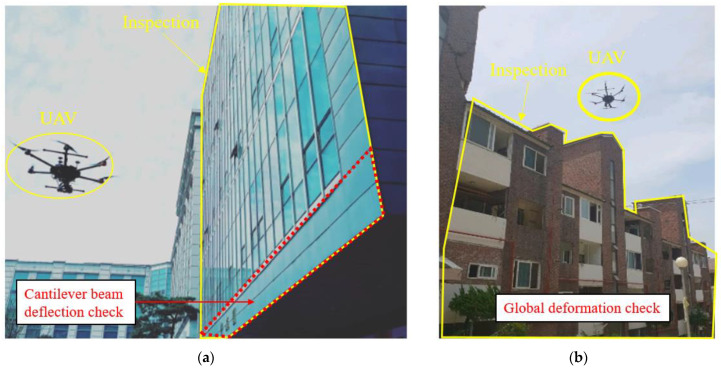
Preliminary safety inspection: (**a**) deflection safety check for cantilever and (**b**) global deformation check using UAV.

This paper introduces the SMART SKY EYE (smart building safety assessment system), which enables rapid preliminary safety inspection of difficult to access areas, using a UAV equipped with a thermal imaging camera and based on crack data obtained through image processing and machine learning in the preliminary stages before on-site safety checks. Figure 1a shows the safety inspection of the cantilever beam on the first floor of a campus building by checking the deflection of the cantilever beam compared with the existing drawing [7]. Figure 1b shows the image of the B-villa, completed in 1996, and the SMART SKY EYE system used to identify global deformation after an earthquake (Magnitude 5.4, 2017).

## 2. SMART SKY EYE

**Figure 2:**
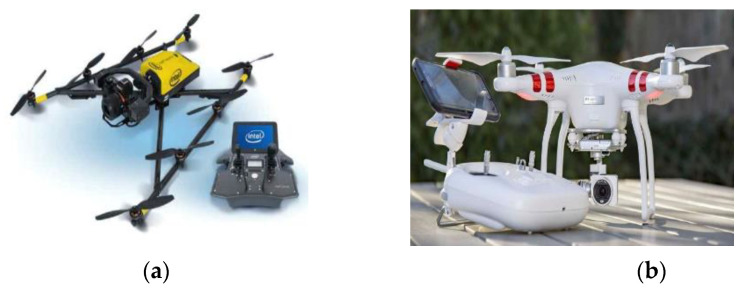
Drones: (**a**) Intel Falcon 8+. (**b**) DJI Phantom 3 Standard.

SMART SKY EYE is a preliminary safety inspection system that can enhance detection efficiency by employing UAVs. With this methodology, defects in structures can be detected and the safety level evaluated before the conventional precise safety inspection. DJI Phantom3 Standard and Intel Falcon 8+ were the UAVs used in this research and are shown in Figure 2.

**Figure 3:**
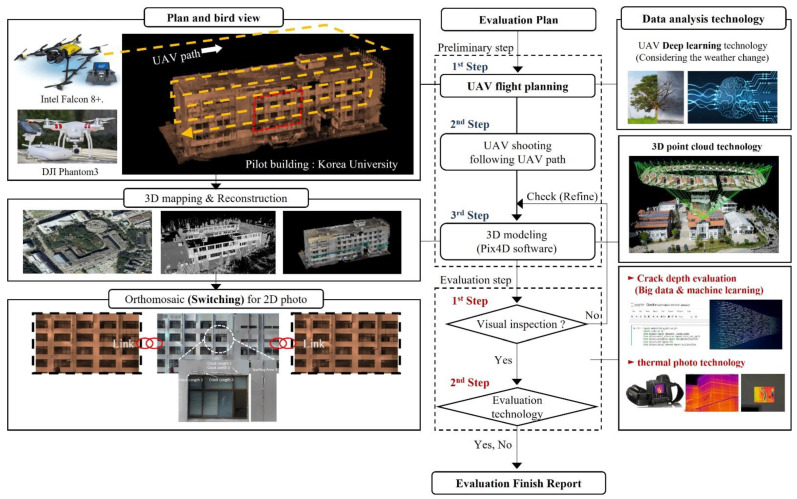
Preliminary safety evaluation method using SMART SKY EYE.

Figure 3 shows the entire process of safety inspection using SMART SKY EYE. The first step is a flight planning to collect information about the structure. Prior flight authorization from the related institutes owing to the Aviation act in Korea and safety planning before flying the UAVs, are considered in this stage. In-flight photography considers the shape, layout, and influence of adjacent buildings. The resolution is regarded as the Ground Sample Distance (GSD) resolved to distance per pixel. The Pix4D automatically calculates the GSD along with the shooting distance, the camera’s focal length, the 3D coordinates of the UAV, etc.

The second stage involves obtaining aerial photography. Aerial photography is performed keeping a certain distance from the building to ensure sufficient target GSD. The flight was taken at slow speeds of less than 1 m/s, and the camera angle was set at approximately 45°. The photographs were taken at many points to have as much overlap between the pictures as possible to aid the subsequent 3D modeling work. In addition, it is important to take the weather conditions into account. Operation with UAVs in rainy weather can lead to hazy images. It is better to avoid filming under cloudy weather or during sunset since insufficient lighting can result in blurry or low-resolution images that can later affect 3D modeling and cause overall problems. In addition, the global positioning system (GPS) received during shooting is essential in providing the hovering of aircraft and location information in 3D modeling. Therefore, it is possible to improve the accuracy of inspection by frequently checking the GPS reception status displayed on the status board. 

The third step involves 3D modeling of the aerial photos using a UAV-specific mapping program. In this paper, a commercial UAV mapping program, Pix4D Mapper, was used. The Pix4D program automatically integrates the spatial information of the photographs, including the UAV location and the shooting angle. A 3D model in the form of a point cloud is obtained through mapping, and an improved model can be obtained by connecting the points through a triangular mesh. In this process, a two-dimensional (2D) orthograph using the 3D model is also obtained to use high-resolution 2D images of each face for detecting the defect. Through this, shape distortion caused by the camera angles can be corrected. 

The post-processing evaluation stage comprises two stages: the first is visual detection based on the high-resolution 2D orthogonality photos and thermal inspection procedure using significant data about crack depths by thermal images with machine learning. If the results of the high-resolution 2D image are not satisfactory to determine a defect, the third step in the preliminary phase to recalibrate the model is repeated by adjusting the resolution (GSD) affecting the image. The visual inspection followed by thermal photo methodology using big data can be used to estimate the cracking sites. In the second stage, thermal imaging techniques are used to determine further the defects of the structure in areas estimated as cracking sites in the first stage, and a report is provided when the evaluation is completed.

### 2.1. Preliminary Implementation Technology

#### 2.1.1. Machine Learning

The parameters that affect the crack temperature were considered as air temperature, humidity, wind speed, and illumination. Decision Tree, Random Forest, AdaBoost, Gaussian Naive Bayes (NB), and Support Vector Machine (SVM) were used to estimate the crack depth using thermal images. Decision trees analyze data and represent patterns that exist between them as predictable combinations of rules in the form of a flow chart-like structure [8]. Creating a Random Forest through multiple decision trees can solve the problem of overfitting. Multiple small decision trees ensemble and determine the largest or average of the final predicted values [9]. 

AdaBoost uses training with sample data step by step; different sample weights are applied to each weak classifier. With a weighted linear combination of multiple weak classifiers, AdaBoost creates a robust classifier that performs better [10]. NB is a statistical classification technique based on Bayes’ theorem, which expresses the probability of an event occurring based on various conditions related to the event. Gaussian NB is one of the most straightforward supervised learning, characterized by using the Bayesian theorem to classify labels according to their features [11]. SVM was proposed to solve the division of space with decision boundaries. The decision boundary determined by the support vectors is the most optimal. The most suitable decision rule generalizes the new sample based on the data [12]. 

**Figure 4:**
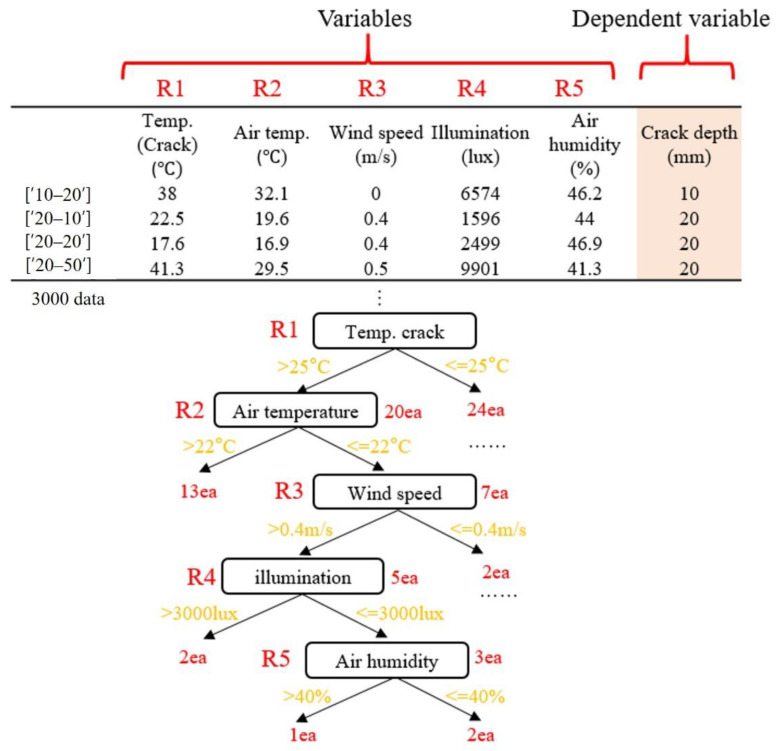
Example of a decision tree algorithm application.

The decision tree algorithm in this paper uses the concept of entropy [13]. The number of cases where each variable affects the total entropy can be expressed, as shown in Equations (1) and (2). The entropy function is introduced to split the branches and to use the algorithm to calculate the information gain for each property. The smaller the gain, the lower the number of cases. The smaller the entropy, the more information can be obtained [13].

(1) $$Entropy\left(A\right)=-\sum_{k=1}^{m}p_{k}\log_{2}\left(p_{k}\right),$$

(2) $$Entropy\left(A\right)=\sum_{i=1}^{d}R_{i}\left(-\sum_{k=1}^{m}p_{k}\log_{2}\left(p_{k}\right)\right),$$

where *R_i_* is the ratio of records belonging to the *i* region after and before the division. For example, suppose a total of 40 data that consider R1 (crack temperature) conditions as shown in Figure 4.

For the given condition, the entropy can be calculated fusing Equation (1) as follows:

(3) $$Entropy\left(A\right)=-\sum_{k=1}^{m}p_{k}\log_{2}\left(p_{k}\right)=-\frac{20}{44}\log_{2}\left(\frac{20}{44}\right)-\frac{24}{44}\log_{2}\left(\frac{24}{44}\right)\approx 0.99,$$

If conditions R1 and R2 are considered, the entropy is calculated using Equation (2) and is given as:

(4) $$\begin{array}{ll} Entropy\left(A\right)= & \sum_{i=1}^{d}R_{i}\left(-\sum_{k=1}^{m}p_{k}\log_{2}\left(p_{k}\right)\right)\\ & =\left(\frac{20}{44}\right)\times \left(-\frac{13}{20}\log_{2}\left(\frac{13}{20}\right)-\frac{7}{20}\log_{2}\left(\frac{7}{20}\right)\right)\\ & +\left(\frac{24}{44}\right)\times \left(-\frac{15}{24}\log_{2}\left(\frac{15}{24}\right)-\frac{9}{24}\log_{2}\left(\frac{9}{24}\right)\right)\approx 0.94 \end{array},$$

The entropy before the split was 0.99; however, after splitting, it reduced to 0.94. Decreasing the entropy by 0.05 means less uncertainty, which means increased purity and information acquisition. Therefore, considering all the conditions from R1–R5, the decision tree division proceeded with machine learning to reduce the overall entropy [13]. The influence of each branched multivariate condition was used as the algorithm for crack area determination, and the Random Forest algorithm was implemented using several decision trees [13]. At this point, the prediction accuracies of the crack depth and adjacent depth (Class) were 72% and 89%, respectively. Therefore, this algorithm could confirm the possibility of crack depth information prediction by using thermal images.

#### 2.1.2. Charge-Couple-Device-Based Image Processing

A charge-coupled device (CCD) pixel-based photogrammetry method, combining image and pixel calculating technology, was used to measure the crack lengths and damaged area of the building. A digital camera is one of the standard inspecting tools in safety checks to record the location of defects and visualize their conditions. With a UAV attached camera, the entire building’s defects can be accounted for rapidly. Additionally, algorithms that quantify the width, size, and length of defects by counting the number of pixels with a constant size improve inspection efficiency and objectivity. In recent years, several studies have been conducted to measure the cracks in concrete with computing technologies, most notably those of image processing. Yamaguchi [14] proposed a method of detecting cracks using processed images by binarizing the brightness.

Henriques and Roque [15] performed an exterior defect inspection of a dam using image-processing algorithms. The aerial photographs of the concrete dam were stitched to generate orthomosaic photos using the software on which image classification was conducted. In a small part of the concrete dam, defects were classified automatically with a commercial classification algorithm. The defect detection was restricted to a small section of the building to prevent misdetection or non-detection of defects. To address such limitations, a pixel-based photogrammetry-based algorithm was used in this study. Due to the limitations of the current recognition technology, the inspector manually detected the defect. 

**Figure 5:**
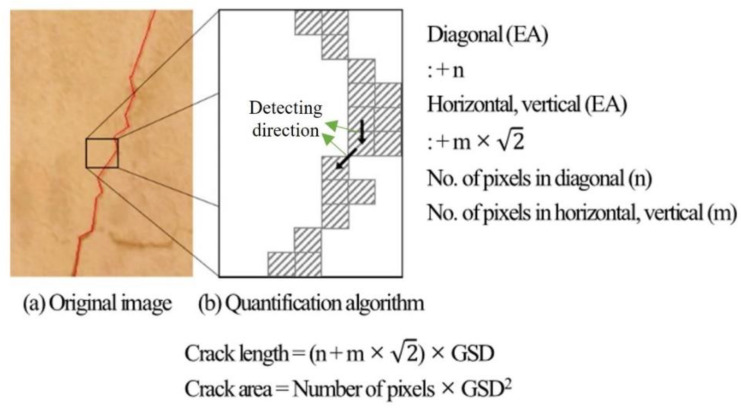
Pixel-based detection algorithm.

First, the investigator selects a defect by clicking along the crack on the orthomosaic. If the crack width is more prominent than 2 mm, an investigator detects the defect by clicking along the center of the cracks. Then, the algorithm draws a line through the clicked pixels by an inspector and calculates the number of pixels included in the cracks. GSD is a fundamental concept suggested in UAV inspection since the model of a camera determines the focal length and represents the real length of pixels in the image. As the length of the GSD pixel is fixed, the algorithm multiplies the number of pixels by the pixel length [16]. The procedure for detecting the defect is manually shown in Figure 5. By calculating the UAV’s short distance and camera resolution, it is possible to determine the pixel size of the image on a real scale.

#### 2.1.3. 3D Point Cloud Modeling

**Figure 6:**
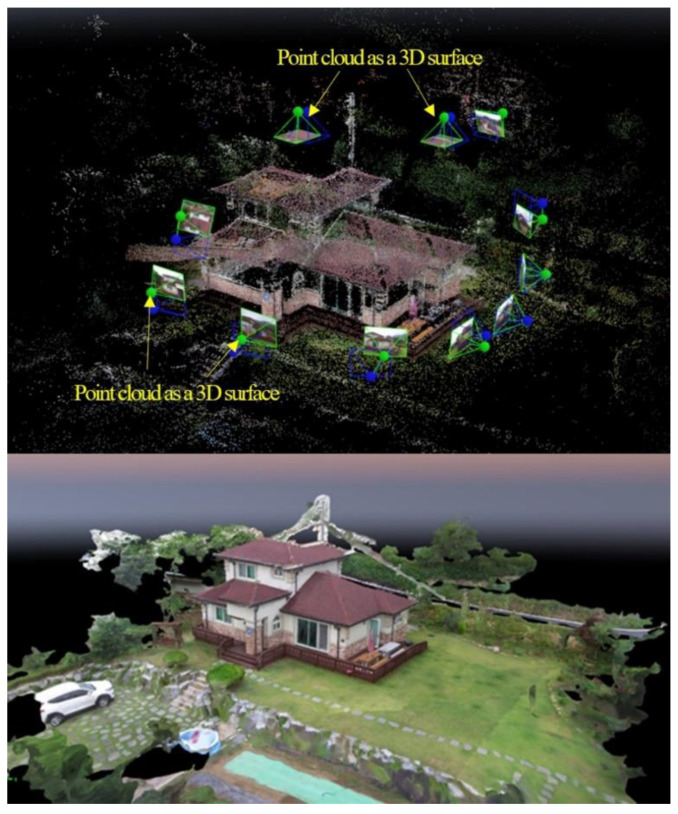
SMART SKY EYE application example of 3D print cloud.

In the 3D point cloud modeling methodology, each of the images captured with the UAV depicts a limited section of the building. The point cloud methodology reconstructs the 3D surface of an object through an alignment of extracting and matching feature points of an object photographed from various angles and represents the point cloud as a 3D surface. In this paper, an image-based architectural exterior 3D point cloud model was created using Pix4D commercial software for point cloud construction and mesh generation. The methodology of realizing 3D location information through a point cloud after shooting a flight using an uncrewed aerial vehicle utilizes the GPS position coordinates and image data acquired at the time of the shooting. This GPS-based survey method allows for the detection of building tilt and vertical displacement measurements and generates high-resolution 3D models (Figure 6).

#### 2.1.4. Thermography

**Figure 7:**
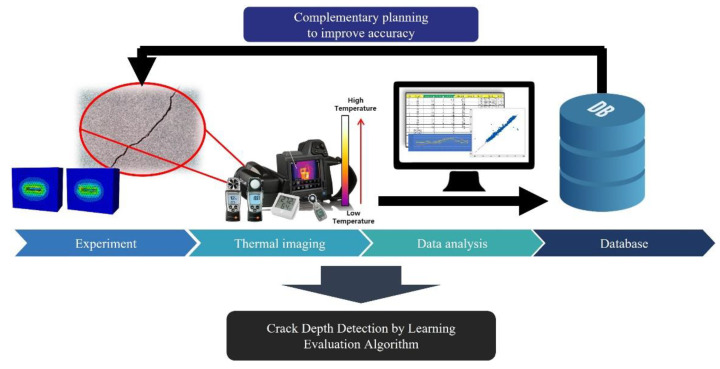
Thermography methodology procedure.

A thermal camera computes the temperature through emissivity, which is a material property. There have been various efforts to apply thermography in building inspection. Aghaei [17] studied a combined system comprising a UAV and thermal camera module. By checking for temperature anomalies in the generator, it was possible to confirm whether the equipment had failed. Tarek et al. [18] explained the methodologies of the unmanned aerial system (UAS)-based thermal imaging practices. An experiment was conducted to assess work empirically, and a UAS-based building inspection method was presented and tested. Figure 7 explains the implementation procedure of the thermography employed in SMART SKY EYE. The experiments performed upon a variety of cracks were conducted using a UAV equipped with a thermal imaging camera. Analysis of the data obtained by the thermal imaging camera was performed using Python programming and statistical analysis tools. The data algorithm was developed to evaluate the crack’s actual depth.

### 2.2. Evaluation Implementation Technology

#### 2.2.1. Displacement Detection

The 3D point cloud model was used to measure the deformation of buildings, such as tilting and vertical displacements. Point clouds were generated from the GPS data of the images recorded by the UAV and represented in a 3D coordinate space with the transformed GPS data. With the locations generated, it was possible to measure the tilting and roof vertex displacement of the building relative to the ground. The edges were calculated as vectors, and the slopes were measured as the angles between the sides and ground. Vertical and horizontal displacements of the edge of the building were estimated through comparisons with each ground point’s 3D coordinate location data. 

The 3D model, generated using the UAV photographed image, uses photogrammetry and GPS to calculate the position coordinate of each point of the point cloud, which can be used for measuring the tilt of the building. For this, a reference point is first set at the lower vertex of the structure to be measured. The vector connecting the reference point and the vertex at the top is called $\underset{x}{\to }$. Next, the base point vectors $\underset{\alpha }{\to }$ and $\underset{\beta }{\to }$ are set by selecting each point at the bottom edge. Compared to the selection of $\underset{x}{\to }$, the selection of the direction vectors $\underset{\alpha }{\to }$ and $\underset{\beta }{\to }$ at the lower part of the building has a higher probability of error due to the selection of points. 

**Figure 8:**
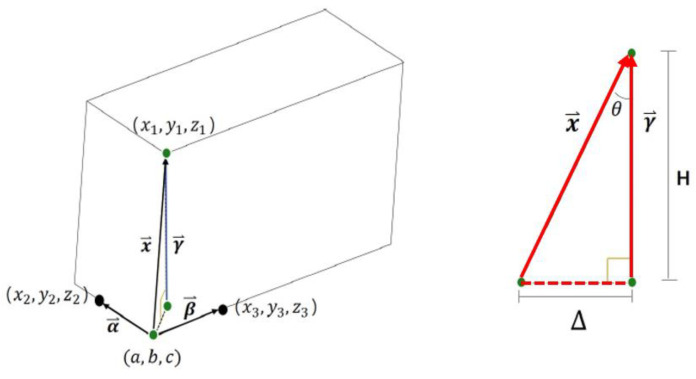
Tilt evaluation procedure.

Therefore, the mean values, over four measurements, are used for calculating $\underset{\alpha }{\to }$ and $\underset{\beta }{\to }$. Then, through the cross product of $\underset{\alpha }{\to }$ and $\underset{\beta }{\to }$, the vector $\underset{\gamma }{\to }$, perpendicular to the plane vector and passing through the upper intersection point, is obtained. The dot product between vectors $\underset{x}{\to }$ and $\underset{\gamma }{\to }$ is used to get *θ* of the two vectors. Since 16 combinations of $\underset{\alpha }{\to }$ and $\underset{\beta }{\to }$ are possible, a total of 16 *θ* values are calculated, and the average is set as the final *θ* (Equation (5)). Then, using the relation between *θ* and the height of the building *H*, the displacement (Δ) between the base and top point of the building is measured. Thus, it is possible to calculate the height and lateral displacement of the upper part of the building and select the level of risk through the evaluation criteria given in Equation (6) (Figure 8).

(5) $$\theta =\sum_{n=1}^{16}\frac{\theta_{n}}{n},$$

(6) $$\tan\theta =\frac{\Delta }{H}=\frac{\sqrt{1-\cos^{2}\theta }}{\cos\theta },$$

#### 2.2.2. Crack Depth Detection

**Figure 9:**
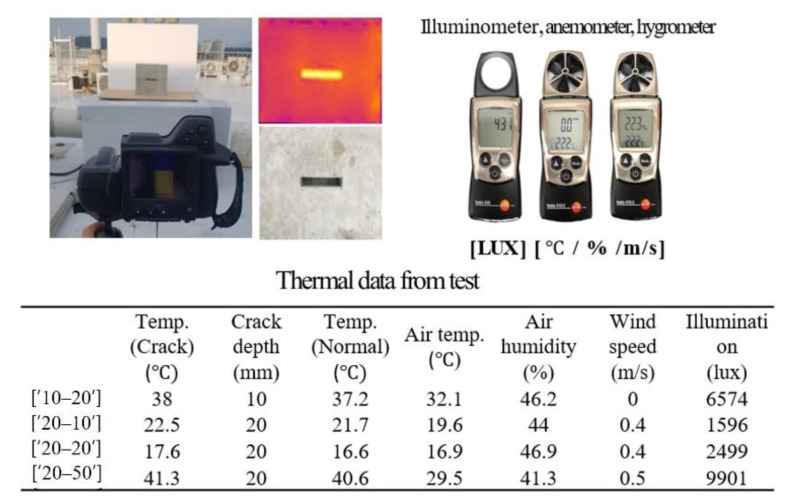
Thermal experiment and test data example.

In SMART SKY EYE, a thermal imaging camera mounted on a UAV was used to detect the fatal crack area in concrete structures. Various shapes of crack specimens, with a depth of approximately 20–80 mm, were fabricated for estimating the depth of cracks over 20 mm (general concrete coating thickness) as these may cause concrete corrosion. The influences of air temperature, humidity, and illumination were selected as parameters (Figure 9). The purpose of the experiment was to estimate a crack depth that can be fatal to the structure at a distance of 5 m to simulate the UAV distance. More than five thousand images were captured during the daytime and late night for one year to observe temperature variations in the crack area of the specimen according to ambient air variables, such as illuminance, humidity, and wind speed. 

Based on the results of the captured thermal image data, we assumed that the temperature capacity caused the differences in the temperature between the crack and the normal portion. Five machine-learning algorithms, namely Random Forest, Decision Tree, AdaBoost, Gaussian NB, and SVM, were used to search for relationships and showed the differences between the mock-up crack depth and crack estimation accuracies as 72%, 61%, 57%, 37%, and 36%, respectively. To improve the accuracy and suggest the appropriate machine-learning algorithms, the number of data must be larger than tens of thousands.

## 3. Application

**Figure 10:**
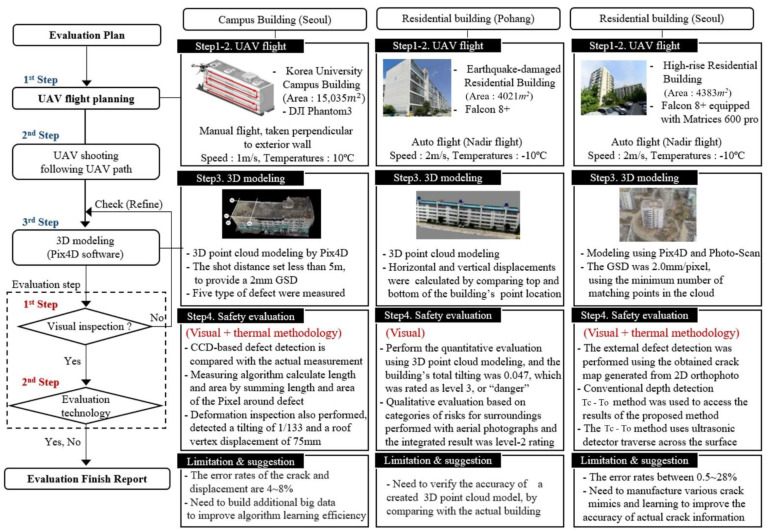
Summary of SMART SKY EYE applications.

Three pilot projects were conducted to test the applicability of the developed method in practical situations (Figure 10). The three cases discussed below used all the technologies of the preliminary and evaluation implementation stages of SMART SKY EYE.

### 3.1. Case 1: Campus Building (Seoul)

**Figure 11:**
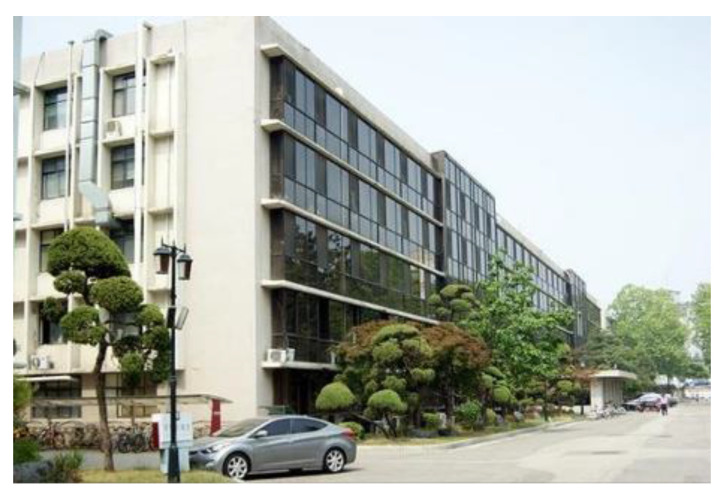
Campus building.

The entire procedure was applied to a campus building (Figure 11) located in Seoul, South Korea. DJI Phantom3 was used for the inspection flight. The construction of the building was completed in 1964. As the building was sufficiently old to have developed exterior defects and is located inside a campus where students congregate, it was appropriate to apply the proposed method. Moreover, there was already an available safety inspection report for this building, against which we could compare the results of the proposed process. 

**Figure 12:**
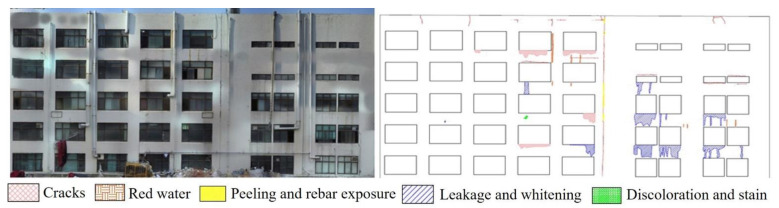
Full-size image and defect map of a campus building.

The total flight time was 6 h; 1681 aerial photographs were captured. The UAV took the pictures while maintaining a vertical direction to the wall of the building. On average, more than 80% overlapping images were taken to model the building. Thirty-six hours were spent on the 3D point cloud modeling by using commercial UAV mapping program, Pix4D. The shot distance was set to less than 5 m to provide a 2 mm GSD. Five types of defect were measured using the 3D point cloud model and the developed pixel-based algorithm. These were reticular cracks (red, 2.543 m^2^), rust traces (brown, 3.02 m^2^), spalling and exposed steel (yellow, 2.9 m^2^), water leakage and whitening (blue, 1.842 m^2^), and discoloration and staining (green, 0.71 m^2^) [19]. The defect map for the campus building is shown in Figure 12.

For evaluating the accuracy of the proposed CCD-based defect detection procedure, particularly the length, width, and crack area measurements, the obtained results were compared with the results of the actual measurement. To measure the building’s exterior defects using tapeline and crack scale equipment, a limited area of the building accessible to the operator was used. The measuring algorithm calculated the length and area by summing the pixel length and area around the defects. The accuracy was measured by comparing the defects identified through the algorithm with that measured directly by the inspector. The error rate of the crack length measurement was less than 4% and was found to increase with the crack length since the algorithm calculates the pixel length linearly. The error rate for crack width measurement was less than 8% for cracks over 2 mm wide but increased drastically when the width of the crack was less than 2 mm. This is because the algorithm calculates the length by adding the length of the pixel and fails to detect defects smaller than the GSD.

A deformation inspection was also performed. As there was a safety report already available for the building, it was possible to compare the results of the report with the proposed method. Based on the obtained location coordinates, the measurements performed on a section of the building detected a tilting of 1/133 (1/141 in the report) and a roof vertex displacement of 75 mm (82 mm in the report). The error rate was 7.19% and 8.53% for the tilt detection and roof vertex displacement, respectively.

### 3.2. Case 2: Residential Building (Pohang)

**Figure 13:**
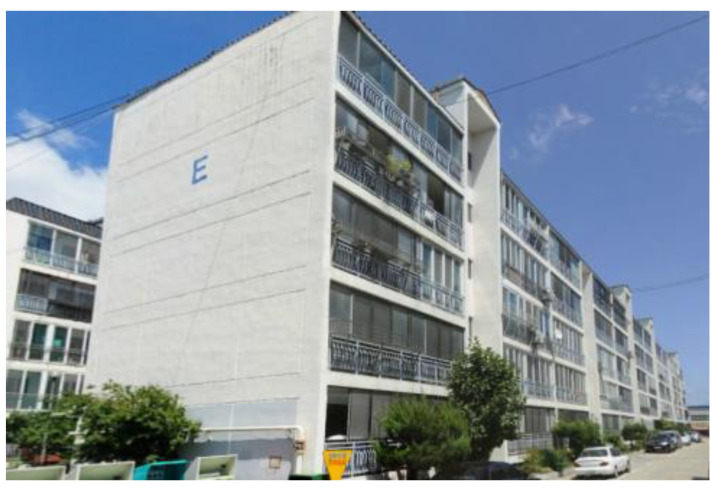
Residential building.

The proposed method was applied to an earthquake-damaged residential building in Pohang (Figure 13). The real flight time for the Intel Falcon 8+ was approximately 40 min, and 1561 pictures were captured with a shot distance of less than 5 m to secure a 2 mm GSD. To assess the damage of the building, the rapid evaluation safety assessment criteria (this is a type of preliminary fast safety inspection to determine the accessibility of a building) were used. Usually, when an earthquake widely affects a city, the evaluation criterion focuses on rapid visual inspection. South Korea’s safety evaluation assessment for earthquake-damaged buildings involves both quantitative and qualitative evaluations.

**Figure 14:**
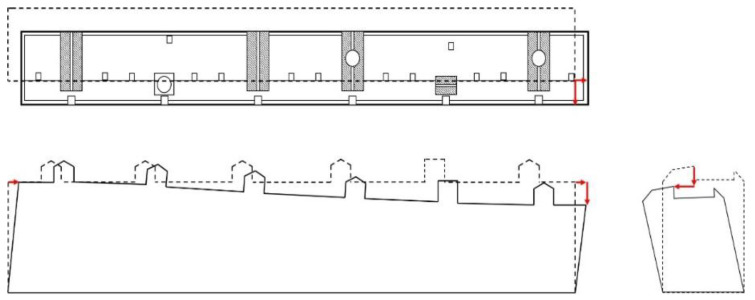
Displacement measurement of the residential building.

3D point cloud modeling was used to perform the quantitative evaluation [19]. Horizontal and vertical displacements were calculated with the proposed method by comparing the top and bottom of the building’s point locations, as depicted in the concept diagram shown in Figure 14. In this study, the location of the bottom of the building was considered stationary through the deformation, whereas the building’s total tilting was 0.047, with the most significant vertical displacement of 420 mm, rated as level 3, or “danger”. 

Qualitative evaluation is based on categories; for example, the risk that the surroundings might collapse or that a facility in the building may fall. This evaluation was performed with the original aerial photographs captured by Intel Falcon 8+. The integrated result was a level-2 rating, or “limited use of the building”. The total result was rated as level 3 (danger) per the rule of selecting the highest risk level.

### 3.3. Case 3: Residential Building (Seoul)

**Figure 15:**
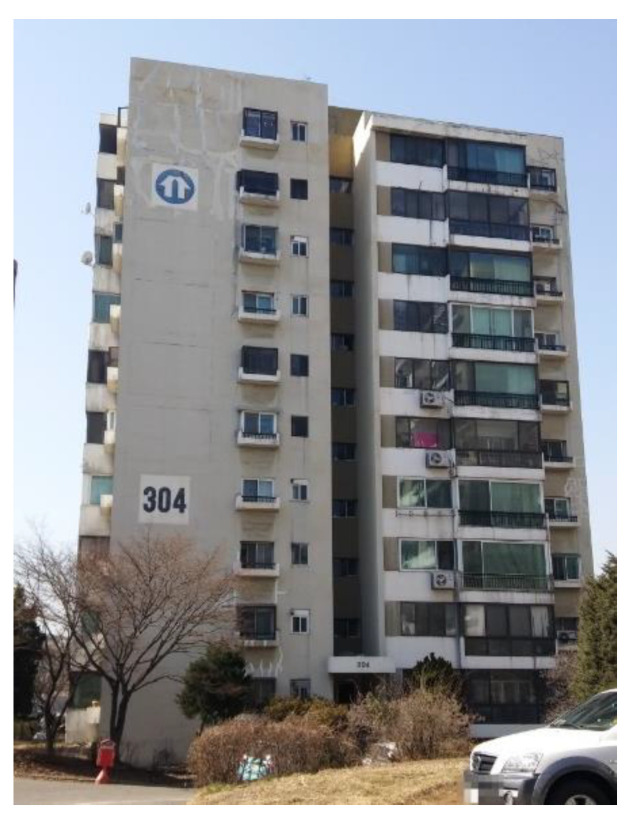
Residential building (Seoul).

Exterior inspection, including a crack depth inspection, was performed on a high-rise residential building (Figure 15), the most common form of an apartment building in South Korea. This building consists of 10 floors of typical box-shaped apartments [20]. The air temperature, humidity, and illuminance were 28 °C, 30%, and approximately 9000 lux. An Intel Falcon 8+ equipped with Matrice 600 Pro was used to photograph the entire facade. The flight path was set the same as the campus building project, taken perpendicular to the exterior wall of the building. After a total time of 3 h spent in the field, 1561 aerial photographs were captured during 1 h of actual flight time. Conventional depth detection equipment, the Tc-To method was used to assess the results of the proposed method. 

The Tc-To method uses an ultrasonic detector to sound the velocity difference in the time taken by an ultrasonic wave to traverse across undamaged and cracked surfaces to measure crack depth. As inspectors cannot approach cracks located higher than 2 m above the ground, three cracks located at the bottom of the apartment were detected and labeled in order as a, b, and c. The conventional method with the ultrasonic detector, measured crack depths for a, b, and c were 80, 43.1, and 55.4 mm, respectively. The crack depth results from a, b, and c, obtained using the proposed defecting crack depth method, were measured as 80, 60, and 60 mm, respectively. The error rates between the conventional and proposed methods, calculated for cracks a, b, and c were 0.5%, 28.17%, and 7.67%, respectively. 

**Figure 16:**
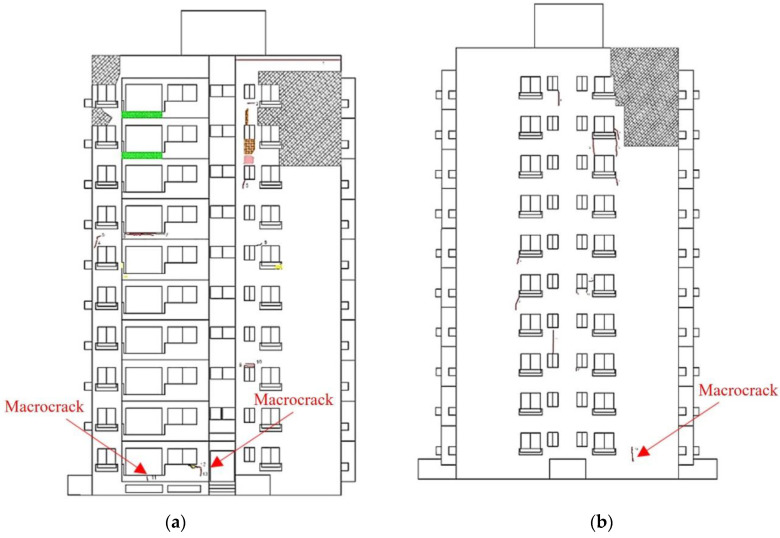

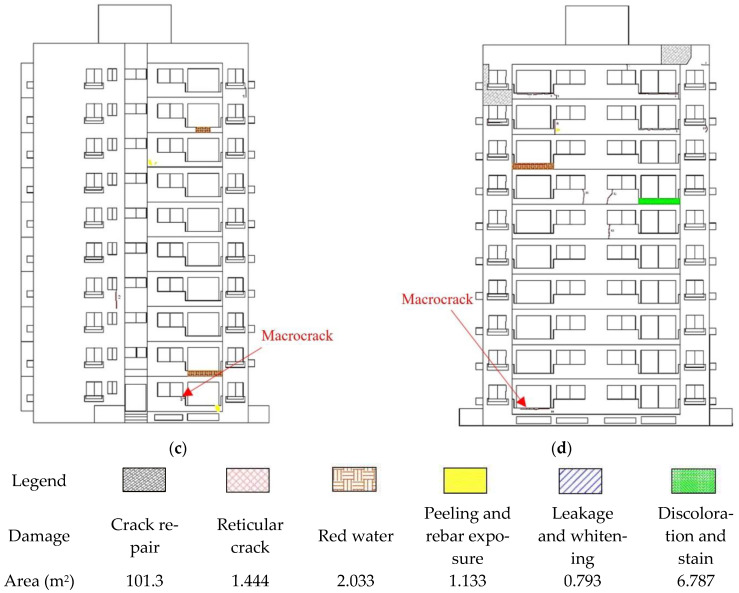
External wall defect detection results: (**a**) front view; (**b**) left view; (**c**) rear view; and (**d**) right view.

Since construction work was about to be started in the apartment, there was limited time to inspect. The bad weather conditions were another reason to set short flight times to shoot aerial photographs. Thus, four walls of buildings with a total area of 2600 m^2^ were photographed over two days, perpendicular to the exterior wall. A total of 491 aerial photographs were captured. In the 3D model, modeling was performed using Pix4D and PhotoScan, both UAV-specific mapping programs. The GSD was 2.0 mm/pixel, and processing required approximately 26 h using the minimum number of matching points in the cloud. After that, point cloud correction was performed to solve the stitching problem caused by low illumination and a low number of photographs. Then, external defect detection was performed using the obtained crack map generated from 2D orthophoto with five macrocracks (Figure 16).

## 4. Conclusions

In response to the fourth industrial revolution, UAVs are being used in various fields of construction. In this paper, the SMART SKY EYE system, which utilizes UAVs for safety inspection procedures, was introduced. This system includes various state-of-the-art technologies, such as UAVs, AI, and thermography, to improve the efficiency and numerical measurements of exterior defect detection procedures, as part of the preliminary safety inspection of building structures. The strength of SMART SKY EYE lies in its capacity to predict the defects of a structure quickly using unmanned aircraft for areas that are difficult to access for humans. Through the proposed technology, improvements in the automation of building maintenance practices can be achieved. Moreover, the objectivity of emergency inspection technologies will be improved, and it will also become easier to track and manage the safety of buildings through the acquired digital data. The following conclusions were obtained as follows:

(1) The SMART SKY EYE system is composed of preliminary and evaluation steps. In the first step, the UAV gathers aerial photographs from the target building by following a flight plan that is optimized to the shape of the building and the weather conditions. 3D modeling is performed using the obtained images. After the preliminary step, an evaluation step is performed. From the 3D point cloud model, a 2D orthomosaic is extracted as a full-scale wall image, and the 3D coordinates of the edges of the building are measured. Finally, preliminary evaluations of the building’s safety are conducted according to the rapid evaluation safety assessment criteria for earthquakes in South Korea.
(2) Three types of inspection technologies were developed and verified. CCD-based crack detection uses the number of pixels and GSD to measure the lengths and widths of cracks. The error rate for the crack length was less than 4%, and this showed an increasing trend with increasing crack length. The crack width error rate was less than 8% for cracks over 2 mm; however, this increased drastically with lengths under 2 mm. Thermography and machine-learning algorithms were applied to detect the crack depths and weld defects. An experiment was conducted to investigate the relationship in the data. The five algorithms used were Decision Tree, Random Forest, AdaBoost, Gaussian NB, and SVM, and these showed accuracy scores of 72%, 61%, 57%, 37%, and 36%, respectively. Furthermore, it is possible to detect weld defects through temperature differences. For measuring the tilt and vertical displacements, 3D coordinates were used as the vectors and the error rates, compared against the report results, which were 7.19% and 8.53%, respectively.
(3) Three pilot applications using UAVs were used to determine the method’s applicability to campus buildings and residential buildings using state-of-the-art techniques from the preliminary and evaluation steps of SMART SKY EYE.
(4) The SMART SKY EYE system obtained aerial photos using a Global Navigation Satellite System. Therefore, there is a possibility of noise caused by GPS reception error during UAV flight. It may be difficult to receive GPS on a cloudy or rainy day because it is sensitive to weather changes. Manual flights were also conducted for areas where the automatic flight was difficult to access in the application projects. However, with manual shooting, it is difficult to maintain a constant flight speed and uniform distance between the UAV and building, and calibration was required through the manual tie point of Pix4D during alignment optimization. In conclusion, we confirmed that the decision of automatic manual flight at the flight plan stage could have a significant effect on the high-resolution point cloud.

## Data Availability

Not applicable.
